# Predator experience enhances giraffe vigilance to oxpecker alarm calls

**DOI:** 10.1186/s12915-025-02395-5

**Published:** 2025-10-10

**Authors:** Anton Baotic, Georgine Szipl

**Affiliations:** 1 Acoustics Research Institute, Austrian Academy of Sciences, Vienna, Austria; 2 Institute for Globally Distributed Open Research and Education (IGDORE), Gruenau im Almtal, Austria

**Keywords:** Alarm call, Anti-predator behaviour, Contextual mutualism, Giraffe, Heterospecific eavesdropping, Oxpecker, Vigilance

## Abstract

**Background:**

Animals often benefit from the alarm calls of other species to detect danger, but how such cues are integrated into vigilance strategies remains unclear. Giraffes (*Giraffa* spp.) rely on early threat detection to avoid ambush and are known hosts of red-billed oxpeckers (*Buphagus erythrorhynchus*), which form mutualistic associations with large mammals by feeding on ectoparasites and emitting alarm calls in response to approaching threats. While these calls are thought to provide early-warning benefits, it remains unclear how giraffes interpret them, and whether their responses vary with prior exposure to predation risk.

**Results:**

We conducted playback experiments across three giraffe populations differing in predator presence to test whether giraffes adjust vigilance in response to oxpecker alarm calls. Individuals in the predator-inhabited reserve maintained vigilance longer than those in predator-free areas, suggesting that prior exposure enhances responsiveness to alarm calls. Acoustic analyses revealed that oxpecker alarm calls are characterized by low harmonic-to-noise ratios, consistent with harsh, broadband signals that are known to enhance attention and urgency perception in alarm contexts. However, call structure alone did not explain vigilance responses; instead responses were modulated by ecological context, specifically whether giraffes lived in areas with or without lions.

**Conclusions:**

Our findings suggest that oxpeckers serve a sentinel-like function and that giraffes use their alarm calls as early-warning signals, with stronger responses observed in populations exposed to predators. This supports the idea that eavesdropping on heterospecific alarm calls can provide context-dependent benefits, with predator-experienced giraffes showing greater sensitivity to oxpecker alarms. By linking behavioral flexibility with ecological context, this study offers a framework for understanding how mutualistic communication systems adapt to changing predation pressures.

**Supplementary Information:**

The online version contains supplementary material available at 10.1186/s12915-025-02395-5.

## Background

Giraffes (*Giraffa* spp.) face significant predation pressure from lions (*Panthera leo*), their primary predator, which target both juveniles and adults across Africa [1]. To mitigate predation risk, giraffes exhibit adaptive vigilance behavior by increasing their alertness in high-risk environments [2]. Despite their exceptional height and keen vision [3], giraffes might rely on additional sensory cues, including vocalizations, to assess threats. Giraffes are known to produce a range of vocalizations, including low-frequency hums [4], hisses and snorts [5]. While the function of hums remains speculative and poorly understood [4], hisses and snorts have been reported in response to potential threats. Hisses in particular have primarily been observed in response to human presence and may signal disturbance [5], although their functional role in antipredator contexts remains to be clarified through experimental research. Many terrestrial mammals integrate multiple sensory modalities in risk assessment, particularly in predator-detection contexts [6]. This raises the possibility that giraffes, like other prey species, may incorporate heterospecific acoustic cues into their vigilance strategies. Given that some African ungulates are known to eavesdrop on alarm calls from sympatric species [7, 8], it is plausible that giraffes also use such information to enhance their predator awareness.

The ability to extract information from the alarm calls of other species, known as ‘eavesdropping’, is a widely observed anti-predator strategy [8–11]. Eavesdropping enhances predator detection without requiring direct encounters and has been documented across a wide range of taxa, including mammals, reptiles and birds, as an adaptive strategy to mitigate predation risk [12–14]. Prey dynamically integrate spatial and temporal variations in predation pressure into their vigilance strategies, balancing threat detection with other essential behaviors such as foraging and social interactions [15]. The 'landscape of fear' model traditionally describes how prey navigate risk by avoiding dangerous areas [16, 17]. However, recent expansions of this framework emphasize that prey adjust vigilance not only in response to spatial risk cues but also based on predictable predator activity cycles and past experiences [18, 19]. Giraffes, despite their elevated visual advantage, might still benefit from heterospecific alarm cues as an additional early-warning system to optimize their vigilance responses.

Oxpeckers (*Buphagus* spp.) are best known for their ectoparasite-feeding behavior on African herbivores [20]. However, their alarm-calling function may significantly influence host vigilance [21–23]. The only empirical study testing oxpecker alarm calls as a vigilance mechanism found that red-billed oxpeckers (*Buphagus erythrorhynchus*) significantly enhanced black rhinos’ (*Diceros bicornis*) ability to detect and evade approaching humans [24]. Red-billed oxpeckers are obligate associates of large African mammals, and are known to produce sharp ‘ksss’ alarm calls when disturbed [25]. Oxpecker calls have been anecdotally reported to alert host animals to potential danger [21, 22] and experimentally shown to serve a sentinel function by alerting black rhinos to approaching humans [24]. However, it remains unclear whether oxpeckers vocalize specifically in response to carnivorous predators, and they are not themselves targeted by large terrestrial predators such as lions or hyenas. Thus, any anti-predator benefits conferred by oxpecker calls likely provide anti-predator benefits to the host as a by-product of the oxpecker’s general disturbance sensitivity, rather than reflecting targeted sentinel behavior. Further research is needed to determine the referential specificity and ecological triggers of oxpecker alarm calls. Oxpeckers are cooperative breeders, with breeding pairs often supported by two to three helpers that contribute to feeding chicks and nest protection [26, 27]. Although they do not coordinate predator detection in a turn-taking manner, as seen in classical sentinel systems like those of meerkats (*Suricata suricatta*) [28], their group-living structure and tendency to vocalize when disturbed may provide functionally similar early-warning benefits to their ungulate hosts. However, only few studies have experimentally assessed their influence on host risk assessment and behavioral decision-making [24]. Oxpecker-host relationships are context-dependent: although oxpeckers feed on ectoparasites, they may also prolong wounds or increase tick burdens in some cases. The relationshp between oxpeckers and their hosts has therefore been characterized as a dynamic mutualistic interaction with a trade-off between potential vigilance benefits and physiological costs [20, 29, 30]. Because giraffes are frequent oxpecker hosts [20, 31], they are likely regularly exposed to oxpecker vocalizations. Such repeated exposure could lead to learned associations between alarm calls and predator presence, particularly in high-risk environments. Unlike many ungulates [32], giraffes do not exhibit a strong group-size effect on vigilance. Instead, scanning behavior appears more sensitive to social context, such as the presence of calves or nearby adult bulls, than to the number of conspecifics per se [33].

Another critical aspect of alarm call efficacy is acoustic structure. Alarm calls vary widely in structure across taxa and can be either tonal or broadband. However, broadband or harsh calls, particularly those with low ‘harmonic-to-noise ratios’ (HNR) are frequently associated with urgency and heightened salience in both mammals and birds [9, 34–37]. Such features are known to heighten vigilance across taxa, but whether oxpecker alarm calls share these properties remains unexplored. If oxpecker calls exhibit low HNR and nonlinearities, they may serve as particularly effective vigilance-inducing cues. Given the variability in oxpecker-host interactions and the context-dependent nature of vigilance adjustments, mutualistic benefits in such systems might not be static but fluctuate based on predation risk, past experiences, and ecological conditions.

We predicted that giraffes in predator-inhabited areas would exhibit prolonged vigilance following oxpecker alarm calls, reflecting experience-dependent risk assessment. To test this prediction, we conducted playback experiments across multiple study sites differing in lion presence, using red-billed oxpecker alarm calls alongside non-alarm control calls of ring-necked doves (*Streptopelia capicola*) and African black-headed orioles (*Oriolus larvatus*). This design allowed us to assess how predator exposure influences behavioral responses to heterospecific vocal cues. To explore potential acoustic correlates of alarm salience, we analyzed HNR of the stimuli. Together, the findings of this study offer insight into how experience and call structure shape vigilance responses in a large herbivore.

## Results

### Oxpecker alarm calls elicit heightened vigilance in giraffes

**Fig. 1:**
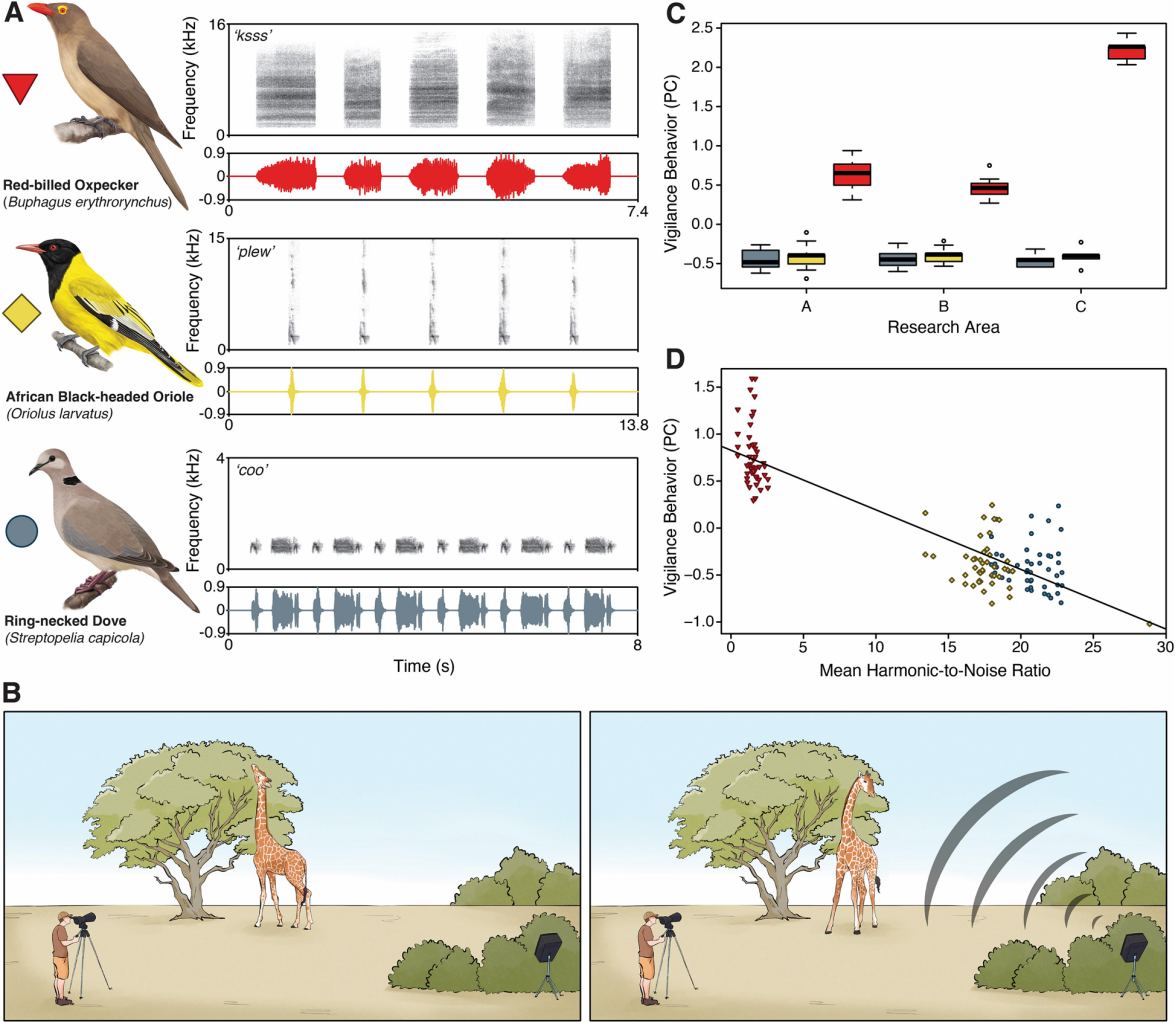
Vigilance responses to oxpecker alarm and non-alarm dove and oriole calls across research areas. **A** Spectrograms and oscillograms of one playback sequence per bird species: five red-billed oxpecker ‘ksss’ calls, five African black-headed oriole ‘plew’, and six ring-necked dove ‘coo’ calls. **B** Experimental setup for playback trials conducted on foot with the focal giraffe feeding stationary at a tree and turning toward the hidden speaker and responding to an oxpecker alarm call. **C** Estimated mean regression scores derived from the LMM of the vigilance behavior component across the all research areas, A and B (lion-free) and C (lion-inhabited), separated by stimulus types: boxplots display the interquartile range (IQR; 1 st to 3.^rd^ quartile), bold lines indicate medians, whiskers extend to the minimum and maximum values within 1.5 times the IQR, and circles mark outliers beyond this range. **D** Mean HNR for individual calls across playback sessions and stimuli types: negative relationship of vigilance behavior and mean HNR of the calls, Regression line: y = −0.063x + 0.828

When presenting 53 giraffes with three stimuli types, i.e., alarm calls from red-billed oxpeckers, and non-alarm ‘plew’ and ‘coo’ calls from African black-headed orioles and ring-necked doves, respectively (for representative examples of the stimuli types see Fig. 1A), giraffes exhibited a range of vigilance behaviors, including not feeding, turning their heads and necks toward the sound source, directing their ears forward, and scanning the surroundings (Fig. 1B). An additional video file shows this in more detail (see Additional file 1).

To quantify these vigilance-related behavioral responses, we performed a principal component analysis (PCA), which summarized the vigilance response durations and total vigilance time into a single’vigilance behavior’ component (see Methods section). The extracted regression scores of this component were used in a linear mixed model (LMM) to test the effect of each stimulus type (‘oxpecker’, ‘dove’, ‘oriole’). The model revealed that giraffes across three research areas (A, B, C) with varying predation presence showed higher regression scores (indicating higher vigilance behavior due to positive loadings on the component; see Methods section) to oxpecker ‘ksss’ calls, compared to dove ‘coo’ and oriole ‘plew’ calls, which is also evident from the strong effect sizes (dove vs. oxpecker: EM ± SE = 1.042 ± 0.215, t = 4.850, CI = 0.618, 1.465; oriole vs. oxpecker: EM ± SE = 1.043 ± 0.208, t = 5.005, CI = 0.632, 1.454; both CIs do not overlap zero). In contrast, dove and oriole calls in comparison produced identically low vigilance responses (EM ± SE = −0.001 ± 0.220, t = −0.005, CI = −0.436, 0.433). Giraffes did not show significant differences in vigilance responses between the two non-alarm control stimuli (dove and oriole), indicating they did not differentiate between these calls. No effects were found for research area, age class, sex, or playback presentation order. Details of the LMM are provided in Additional file 2: Table S1.

### Predation risk enhances vigilance responses to oxpecker alarm calls

Giraffes in all research areas responded more strongly to oxpecker alarm calls compared to non-alarm control stimuli, the magnitude of this vigilance response varied across the research areas. This pattern reflects the strong interaction between stimulus type and research area. Specifically, giraffes in area C (lion-inhabited) exhibited significantly stronger vigilance responses to oxpecker alarm calls than those in the predator-free areas A and B (Area C vs. A: *P* = 0.0001; Area C vs. B: (*P* < 0.0001) (see Table 1, Fig. 1), while responses to oxpecker calls did not differ between areas A and B (*P* = 0.7022). In contrast, vigilance responses to dove and oriole calls did not differ across research areas (*P* > 0.99), indicating that predation context selectively influenced vigilance responses to oxpecker alarm calls but not to non-alarm stimuli. This supports growing evidence that mammals fine-tune their anti-predator responses based on learned associations between alarm signals and actual predation threats [38].

**Table 1:** Comparison of vigilance responses to different stimulus types (oxpecker, dove, oriole) across research areas (A, B, C). Pairwise contrasts derived from the LMM for the interaction between stimulus type and research area. Results are averaged over age class, sex, and stimulus presentation order. P values were adjusted using Tukey’s test

Research area	Stimulus Type ‘Dove’			
	**Estimated mean**	**SE**	**t ratio**	***P* value**
A vs. B	−0.021	0.246	−0.084	0.9962
A vs. C	0.040	0.369	0.108	0.9936
B vs. C	0.061	0.366	0.165	0.9851
**Research area**	**Stimulus Type ‘Oriole’**			
	**Estimated mean**	**SE**	**t ratio**	***P* value**
A vs. B	−0.011	0.24	−0.046	0.9988
A vs. C	0.005	0.387	0.013	0.9999
B vs. C	0.016	0.395	0.04	0.9991
**Research area**	**Stimulus Type ‘Oxpecker’**			
	**Estimated mean**	**SE**	**t ratio**	***P*** **value**
A vs. B	0.190	0.236	0.802	0.7022
A vs. C	−1.495	0.342	−4.368	0.0001
B vs. C	−1.684	0.346	−4.873	< 0.0001

### Lower mean harmonic-to-noise ratio increase vigilance, modulated by predation context

Based on 26 playback sequences per species, our acoustic analysis confirmed that oxpecker calls exhibited the lowest mean HNR (mean ± SD = 1.63 ± 0.44, N_calls_ = 130), compared to oriole (17.66 ± 2.71, N_calls_ = 130) and dove (20.82 ± 1.46, N_calls_ = 156) calls, reinforcing their distinct acoustic structure. By investigating the effect of mean HNR on the regression scores of the ‘vigilance behavior’ component, the LMM revealed a strong negative effect of mean HNR, indicating that giraffes exhibited greater vigilance when exposed to calls with lower HNR (EM ± SE = −0.055 ± 0.010, t = −5.599, 95% CI = 0.074 to −0.036, Fig. 1D). This supports prior research demonstrating that calls with noisier, less tonal structures enhance vigilance responses by increasing signal urgency [9, 35]. Furthermore, vigilance behavior scores were higher in research area C compared to areas A and B. Specifically, vigilance was higher in area C versus area A (EM ± SE = 1.563 ± 0.350, 95% CI = 0.872 to 2.253) and area C versus area B (EM ± SE = 1.782 ± 0.354, 95% CI = 1.083 to 2.480), with no difference between areas A and B. No effects were found for age class, sex, and stimulus presentation order, indicating that vigilance responses were amplified by low HNR of oxpecker alarm calls and predation context. Details of the LMM are provided in Additional file 2: Table S2.

When analyzing the interaction between mean HNR and research area, we found a strong effect showing that vigilance responses to lower HNR values were strongest in area C compared to area A and B (cp. Table 2). Importantly, mean HNR did not differ between research areas (Kruskal–Wallis H test: χ^2^ (2) = 0.0963, *P* = 0.953), confirming that the observed differences in vigilance behavior were not caused by variations in the acoustic properties of the playback stimuli. The effect of mean HNR on the vigilance behavior component was significantly stronger in area C, which was the only site with large predators, compared to area B (*P* = 0.0204). However, while vigilance responses to lower HNR values were also higher in area C compared to area A, this effect did not reach the conventional significance threshold (*P* = 0.0651), suggesting a potential trend. This result highlights that HNR alone does not fully explain the heightened vigilance responses observed in area C. Instead, it suggests that giraffes possess a predisposed sensitivity to oxpecker alarm calls, but the duration and magnitude of their vigilance responses may be further shaped by prior exposure to predation risk [38, 39].

**Table 2:** Pairwise contrasts for the interaction (‘:’) between mean harmonic-to-noise ratio (HNR) and research area. Results are averaged over age class, sex, and stimulus presentation order. P values were adjusted using the Tukey method for multiple comparisons

Contrast	Estimated mean	SE	t ratio	*P* value
Mean HNR: A vs. B	0.102	0.151	0.675	0.7790
Mean HNR: A vs. C	−0.506	0.223	−2.273	0.0651
Mean HNR: B vs. C	−0.607	0.222	−2.739	0.0204

### Acoustic comparison between oxpecker ‘ksss’ calls and giraffe hisses

Giraffe hisses and oxpecker alarm calls differed significantly in both ‘peak frequency at maximum amplitude’ (fpeak) and duration. Wilcoxon-Mann–Whitney U tests (MWU) revealed that oxpecker calls had a significantly higher fpeak (mean ± SD = 4993 ± 1216 Hz; Min–Max = 2406–6535 Hz) than giraffe hisses (689 ± 612 Hz; Min–Max = 140–2320 Hz; MWU: Z = −5.68, *P* < 0.0001). Oxpecker calls were also longer in duration (0.90 ± 0.15 s; range: 0.608–1.114 s) compared to giraffe hisses (0.72 ± 0.22 s; Min–Max: 0.238–1.038 s; MWU: Z = −2.84, *P* = 0.0045).

### Reaction speed to alarm calls is consistent, but predator presence prolongs vigilance

In addition to measuring durations of vigilance-related behavioral responses, we analyzed response latencies to determine how quickly giraffes responded to different stimuli. Specifically, we examined the latency of not feeding and turning head and neck toward the speaker, as these variables represent key initial vigilance behaviors in response to potential threat. Response latencies varied significantly between stimulus types (Kruskal–Wallis H test: not feeding: χ^2^ (2) = 81.078, *P* < 0.001; turn to: χ^2^ (2) = 91.18, *P* < 0.001), with significantly faster reaction times in response to oxpecker calls compared to both dove and oriole calls (see Table 3). Post hoc Dunn’s tests confirmed that giraffes responded significantly faster to oxpecker calls than to dove and oriole calls for both latency measures (all adjusted *P* < 0.001; Table 3). No significant differences were observed between dove and oriole calls, indicating that only oxpecker alarm calls elicited an immediate response. However, response latencies did not differ across research areas (not feeding: χ^2^ (2) = 0.354, *P* = 0.838; turn to: χ^2^ (2) = 1.862, *P* = 0.394), indicating that predation risk did not influence the speed of response. Instead, response latencies appeared to be primarily driven by stimulus type, likely reflecting the heightened sensitivity to oxpecker alarm calls due to their broadband, chaotic acoustic structure. These results suggest that while giraffes ‘ responses in research area C were longer when hearing oxpecker calls, their initial reaction speed remained consistently rapid across all research areas, regardless of predation risk.

**Table 3:** Dunn’s post hoc tests for response latencies (not feeding, turn to speaker) across playback stimuli types. Sample sizes per group are indicated by N₁_,_₂ and reflect the number of individuals tested with each stimulus type: oxpecker (*n* = 26), oriole (*n* = 25), and dove (*n* = 22); not all giraffes received both control stimuli. Z statistics and 2-sided *P* values with Bonferroni adjustment are shown. Bold values indicate significant differences after controlling for multiple testing

Pairwise comparison	N_1,2_	Z statistic	Adjusted P
**Not feeding**			
Dove – Oriole	22,25	0.0	1.0
Dove – Oxpecker	22,26	7.72	** < 0.0001**
Oriole – Oxpecker	25,26	7.76	** < 0.0001**
**Turn to**			
Dove – Oriole	22,25	0.24	1.0
Dove – Oxpecker	22,26	8.31	** < 0.0001**
Oriole – Oxpecker	25,26	8.10	** < 0.0001**

## Discussion

### Oxpecker alarm calls and predation risk elicit heightened vigilance in giraffes

Animals must continuously adapt their vigilance strategies to balance foraging efficiency with predator avoidance, often integrating social and environmental cues to optimize risk assessment [15]. Our findings demonstrate that giraffes incorporated oxpecker alarm calls into their vigilance behavior, even in the absence of direct visual confirmation of threats.

Giraffes in our study selectively responded to oxpecker alarm calls, filtering out non-alarm dove and oriole calls. Because we tested only solitary giraffes, these responses are unlikely to be driven by social facilitation, strengthening the interpretation that vigilance was triggered by the alarm calls themselves. This indicates that giraffes may integrate learned heterospecific cues into their risk assessment strategies. Such selective attention to alarm calls aligns with evidence from other prey species, where associations between heterospecific alarm calls and predator presence are shaped by experience [7, 40]. In this context, oxpecker calls may serve as indirect risk cues, enabling giraffes to anticipate danger and respond proactively. Similar patterns were observed in wildebeests, which prioritize zebra calls over impala calls in predator-rich landscapes [7], and in Gunter’s dik-diks (*Madoqua guentheri*), which eavesdrop on white-bellied go-away birds (*Corythaixoides leucogaster*) for early-warning benefits despite their solitary nature [40]. This prioritization of reliable cues aligns with predictions from signal detection theory, which proposes that prey optimize vigilance by responding to signals that maximize the trade-off between accurate threat detection and minimizing false alarms [10, 41].

Giraffes in the predator-inhabited area exhibited longer vigilance durations than those in the predator-free areas. This finding is in line with the concept of ‘dynamic landscapes of fear’ [18] and supports previous studies that vigilance plasticity is shaped by prior risk exposure and learned predator associations [7, 38, 42, 43]. By incorporating learned information about predator activity and the reliability of heterospecific alarm calls, giraffes may fine-tune their responses to maximize survival benefits while minimizing unnecessary vigilance costs.

Prior studies have found that vigilance and stress hormone levels in prey species (impala, blue wildebeest, and zebra) did not significantly differ between reserves with and without large predators—with research area C previously classified as a predator-free environment in these analyses [42, 44]. These findings suggest that predator absence alone is not necessarily associated with lower vigilance levels, nor does predator presence always enhance them. Instead, vigilance appears to be shaped by a combination of direct predator encounters and experience. Since lions were recently introduced to research area C, with confirmed giraffe predation events [39], giraffes in this region might have developed a heightened sensitivity to oxpecker alarm calls as a result of an arising predator exposure. Similarly, studies on kudu and zebras have shown that vigilance at waterholes increases in response to predator presence but decreases when heterospecifics are nearby, suggesting that mixed-species groups contribute to shared risk assessment [45]. Our findings suggest that giraffes, like other ungulates, modify vigilance responses based on spatial risk and heterospecific alarm reliability, integrating both direct and indirect threat cues [7, 38].

Our acoustic analysis revealed that oxpecker alarm calls exhibit low ‘harmonic-to-noise ratios’ (HNR) and ‘deterministic chaos’, features known to enhance attention and sustain vigilance [34–36]. Alarm calls with nonlinear acoustic structures have been shown to capture receiver attention and prevent habituation as shown for example in meerkats (*Suricata suricatta*) [46] and red deer (*Cervus elaphus*) [47], where harsh, nonlinear signals effectively trigger vigilance responses. In red deer, harsh roars that include chaotic elements and sudden amplitude changes increased attention in receivers and helped prevent habituation, suggesting that unpredictability in acoustic structure enhances signal salience [47]. In meerkats specifically, naturally occurring nonlinear vocal phenomena, such as subharmonics, embedded in alarm calls evoke stronger anti-predator responses than structurally matched linear calls. Individuals exposed to these calls show heightened vigilance, reduced foraging, and an increased likelihood of fleeing to safety, reinforcing the idea that acoustic unpredictability can amplify urgency perception [48]. Similarly, Massenet et al. (2025) reviewed how nonlinear acoustic phenomena, including chaotic structure and spectral roughness, occur widely in animal vocalizations and are often associated with increased arousal and urgency. These features are thought to enhance signal salience and amplify behavioral responses in receivers, which supports a general function for nonlinearity in risk communication across taxa [49]. Taken together these findings support the idea that giraffes perceive oxpecker alarm calls as generalized risk cues, primarily due to their acoustic properties (harshness and nonlinearity), rather than as referential signals specifying predator identity. In contrast, giraffes did not show differences in their response to control stimuli (dove and oriole), indicating that they selectively responded to the oxpecker calls. This lack of differentiation between control stimuli supports the interpretation that only oxpecker alarm calls convey reliable information about potential risk. Following the playback experiments, we conducted an exploratory acoustic analysis to assess whether specific call features could account for observed behavioral patterns. Control species (dove and oriole) were chosen based on their local presence and ecological relevance, not on acoustic structure. While they happen to produce tonal calls with high HNR, this was not a selection criterion, but allowed us retrospectively to examine how signal structure may relate to giraffe responses. Although we did not experimentally manipulate the acoustic structure of the used stimuli, the observed associations between lower HNR and increased vigilance suggests that acoustic harshness may influence how strongly alarm calls are perceived and responded to.

Our findings indicate that acoustic structure alone does not fully account for vigilance duration, as responses were modulated by predation risk and prior exposure to predators. The stronger significance of the oxpecker stimulus type (A vs. C, Table 1) compared to the effect of mean HNR alone (A vs. C, Table 2) suggests that vigilance responses were likely influenced by other acoustic properties of oxpecker calls, beyond just HNR. Features such as frequency, modulation, or temporal patterns may also contribute to how giraffes assess alarm cues, with noisier structures (i.e., lower HNR) acting as one element within a broader vigilance-eliciting mechanism. Alternatively, in high-risk environments like area C, where lions are present, giraffes may already maintain an elevated baseline vigilance, reducing the relative influence of acoustic stimuli—a potential ceiling effect [50]. These findings highlight that both the acoustic structure of alarm calls and environmental context (predation risk) jointly shape giraffe vigilance responses.

Across all research areas, giraffes responded quickly and consistently to oxpecker alarm calls, indicating a predisposed sensitivity to these cues. However, the duration of vigilance bahviour was significantly longer in the predator-inhabited area, suggesting that predator presence may contribute to prolonged scanning behavior. Nevertheless, we cannot rule out the influence of other site-specific factors that might also shape vigilance patterns. This suggests that while the initial response to alarm calls is reflexive, the decision to remain vigilant is modulated by experience and perceived risk. Rapid responses to alarm calls are widespread across taxa and are often shaped by selection for fast, reliable threat detection [51]. Birds, for example, can react to alarm calls in under 100 ms, highlighting strong selection for immediate responses to urgent threats [51]. However, vigilance duration is more flexible, with prey adjusting their time spent scanning based on perceived danger. Research in caribou has demonstrated that individuals fine-tune their vigilance responses as they gain experience [19]. Similarly, our results indicate that giraffes react quickly to oxpecker alarms but adjust vigilance duration depending on risk exposure, supporting the role of learning and integrating past predator encounters and recource availability into their decision-making processes [52].

Although apparent structural similarity between oxpecker alarm calls and giraffe hisses (see spectrograms in [5]) might imply that giraffes responded to oxpecker alarm calls because these calls resemble their own vocalizations, our data suggest that this is unlikely. A quantitative comparison of both call types showed that oxpecker calls had substantially higher fpeak values and were longer in duration than giraffe hisses. These differences were statistically significant, indicating that the calls are structurally distinct in these key acoustic features. While only experimental tests could definitively determine whether giraffes perceive these calls as acoustically similar, our acoustic comparison suggests that the calls are acoustically distinct. Given these findings, the observed vigilance responses are unlikely to be driven solely by acoustic resemblance. Instead, our findings indicate that giraffes responded to an ecologically relevant heterospecific cue that reliably signals risk. The fact that only giraffes in lion-inhabited areas exhibited longer vigilance responses (despite all populations being exposed to the same stimuli) suggests that ecological experience underlies the observed behavior. Similar patterns have been documented in other taxa: for example, Diana monkeys (*Cercopithecus diana*) respond differently to alarm calls produced by Campbell’s monkeys (*C. campbelli*) depending on the predator being signaled, even though these calls are not produced for them [53]. Likewise, black-casqued hornbills (*Ceratogymna atrata*) increase their vigilance when hearing primate alarm calls associated with predators, indicating learned recognition of heterospecific cues without any acoustic similarity to their own vocal repertoire [54]. These examples illustrate that receiver responses to alarm calls can emerge through ecological relevance and experience, rather than requiring structural resemblance to the listener’s own calls.

Collectively, our findings suggest that giraffes integrate acoustic properties, ecological context, and past experience to optimize their vigilance responses to oxpecker alarm calls. While aspects of alarm call structure, particularly harshness linked to deterministic chaos as a form of nonlinear vocal production [36] may enhance initial detection, vigilance duration appears to be context-dependent, and shaped by learned risk assessment rather than acoustic features alone.

### Contextual mutualism: a dynamic framework for heterospecific alarm responses

Our findings illustrate a case of context-dependent interaction within a broader mutualistic relationship. Red-billed oxpeckers and giraffes are well known to engage in mutualism, with giraffes providing feeding and transport opportunities and oxpeckers removing ectoparasites [29]. However, the alarm call interaction we observed may function more as a commensal benefit to giraffes: while giraffes gain early-warning information, oxpeckers may not derive a direct benefit from alerting their hosts. Such asymmetric outcomes are not uncommon and often occur within facultative or conditional mutualisms [55–57]. We interpret our results through the lens of ‘contextual mutualism’, whereby the benefit giraffes derive from oxpecker alarm calls varies with ecological risk. Giraffes in the predator-inhabited area sustained vigilance longer following alarm calls, indicating a higher perceived utility of these cues under elevated threat. In contrast, individuals in predator-free areas resumed foraging more rapidly, suggesting a reduced reliance on heterospecific alarm signals when predation risk is low. In predator-free environments, the value of alarm calls might diminish, and if the physiological costs of ectoparasite feeding or wound aggravation outweigh the protective benefits, giraffes might become less tolerant of oxpecker presence. Such shifts in cost–benefit balance are consistent with theoretical predictions that mutualisms can become neutral or even antagonistic when environmental conditions no longer favor cooperation [55, 58]. This conditional responsiveness supports a broader view of mutualisms as flexible, context-dependent interactions shaped by local ecological pressures and species experience [59]. While oxpeckers are not classical social sentinels, their alarm calls may serve a sentinel-like function by providing reliable early-warning cues to giraffes in high-risk landscapes. Similar dynamics occur in other eavesdropping systems, such as Gunter’s dik-diks responding to go-away bird alarms, where nonsocial species gain survival benefits by monitoring heterospecific risk cues [40]. A comparable context-dependent dynamic is observed in cleaner fish systems, where cleaners consistently benefit from host access, but the value to hosts fluctuates with parasite burden. Hosts engage more frequently with cleaners when parasite loads are high, indicating that the mutualistic value is driven by ecological need [60]. However, cleaner fish also exhibit cheating behavior by feeding on host mucus, tissue, and occasionally blood [61]. Similarly, oxpeckers may increase blood-feeding behavior under low tick abundance, reducing the benefit to hosts [29]. While oxpecker alarm calls are possibly not directed at giraffes, these vocalizations may provide unintentional but reliable early-warning cues. This illustrates how mutualistic interactions can include asymmetric, context-dependent benefits, consistent with the concept of conditional outcomes in mutualism [55]. Our findings thus demonstrate a case of contextual mutualism, in which giraffes experience greater benefit from the interaction in predator-rich environments.

### Conservation implications and ecological relevance

Beyond their role in early-warning systems, oxpeckers provide additional ecological benefits by reducing ectoparasite loads, contributing to host health and fitness [29]. Our results demonstrate that giraffes adjust their vigilance behavior in response to oxpecker alarm calls, particularly in predator-inhabited landscapes, reinforcing the ecological relevance of these interspecies interactions. The dual role of oxpeckers (as ectoparasite removers and as incidental sentinels) suggests a multifaceted contribution to host well-being and risk assessment. As such, the decline of oxpeckers in human-altered habitats [62] could impair both parasite management and anti-predator vigilance behaviors in giraffes. These findings underscore that heterospecific alarm communication may serve a compensatory function in species like giraffes, which often form loose and variable group structures [63]. In anthropogenic environments where social vigilance is reduced, reliance on heterospecific cues may become even more critical. Moreover, given that giraffes initially increase vigilance in response to human disturbance but later habituate [64], persistent anthropogenic exposure might alter how they process alarm cues over time, potentially desensitizing them to biologically relevant indicators of predation risk. Playback experiments with naïve individuals could determine whether giraffes are predisposed to respond to oxpecker alarms or if these vigilance behaviors are shaped primarily by experience. Additionally, longitudinal studies tracking how sensitivity to alarm calls fluctuates over time in response to environmental changes or predator reintroductions could provide deeper insights into how mutualistic relationships persist under changing conditions. By anchoring our conservation perspective in observed differences in alarm-call responsiveness, we highlight the importance of preserving mutualistic relationships that enhance adaptive risk assessment in dynamic ecosystems.

## Conclusions

These findings underscore the complex interplay between acoustic signal properties, environmental context, and prior experience in shaping giraffe vigilance behavior. By demonstrating that giraffes exhibit consistent yet modulated responses to oxpecker alarm calls, this study highlights how socially flexible, visually oriented herbivores integrate auditory cues into adaptable anti-predator strategies. The interaction between call harshness and predator exposure offers novel insight into how mutualistic alarm systems operate under varying ecological pressures. Framed through the lens of ‘contextual mutualism’, our results suggest that the functional value of alarm calls is not fixed, but dynamically shaped by local predation risk and the giraffe’s prior experience. By linking behavioral flexibility with ecological context, this study offers a grounded perspective on how mutualistic signals function across varying environmental conditions.

## Methods

### Research areas

We studied wild Southern giraffe populations across seven privately owned reserves in the Limpopo Province of South Africa, during July–September 2023 and July–September 2024. These reserves were grouped into three research areas (A, B, and C) based on geographic proximity. Area A included five reserves lacking large predators such as lions, while area C had lions introduced between 2019 and 2020. Area B (Mogalakwena) remained lion-free. Red-billed oxpeckers (*Buphagus erythrorhynchus*) were present and regularly observed interacting with giraffes across all three study areas, confirming that giraffes at each site had ecological exposure to the heterospecific alarm cue used in the playback experiments (Table 4).

**Table 4:** Overview of study sites grouped into research areas A, B, and C based on geographic proximity and predator presence. Giraffe sample sizes, age distribution, and reserve sizes, and oxpecker presence are shown. Average inter-reserve distances reflect site clustering in Area A and spatial separation from Areas B and C. Lions were reintroduced to Lapalala Wilderness (Area C) between 2019 and 2020, resulting in confirmed giraffe predation events by 2024 [39]

Research area	Reserve	Size (ha)	Individuals (N)	Adult	Sub-adult	Calf	Predator presence	Oxpecker presence	Inter-reserve distances (avg. km)
A	Zebula Golf Estate and Spa	1600	2	2	-	-	No large predators (lion-free)	Present	13.5 km between the 5 reserves within research area A
	Adventures With Elephants (adjacent to Zebula)	300	11	7	2	2			
	Zwartkloof Private Game Reserve Estate	2031	1	-	-	1			
	Mabalingwe Nature Reserve	8500	4	3	-	1			
	Safari Plains	12,000	8	6	1	1			
B	Mogalakwena River Reserve	1500	20	18	1	1	No large predators (lion-free)	Present	~ 249 km from Area A
C	Lapalala Wilderness	48,000	7	5	1	1	Seven lions introduced (2019–2020); Fifteen lions present by September 2024	Present	~ 112.7 km from Area A; 15 lions present by September 2024

### Distribution and ecological relevance of playback species

**Fig. 2:**
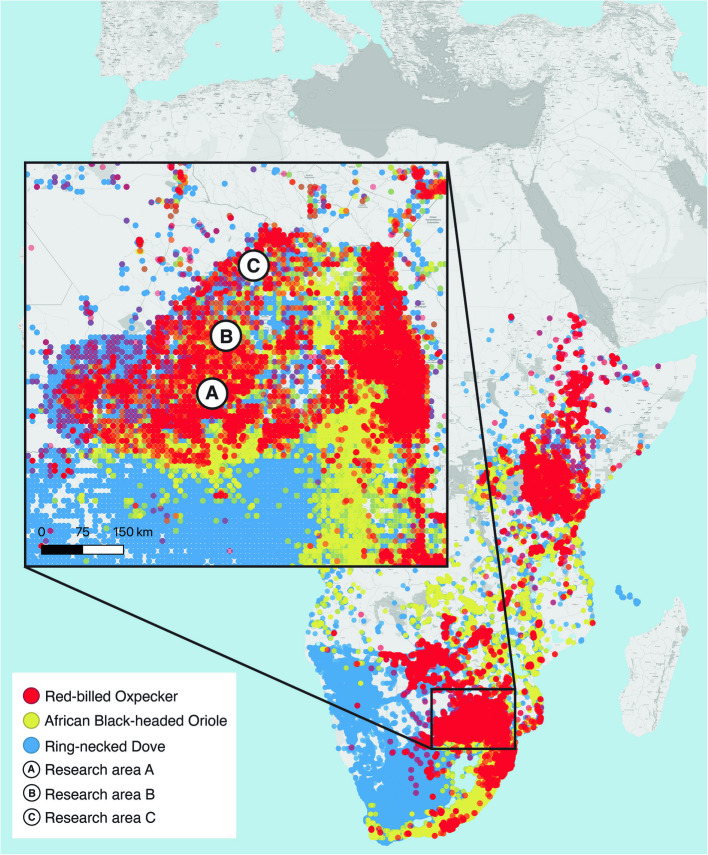
Geographic distribution of the red-billed oxpecker, African black-headed oriole, and the ring-necked dove across the research areas A, B, and C in Limpopo Province, South Africa. All three species were present at all study sites, confirmed through direct observation and Global Biodiversity Information Facility (GBIF) records. The map visualizes species occurrence across the three research areas using GBIF data [67]. The map was created using QGIS version 3.40.3

We selected bird species that are wiedespread and native to the Limpopo biome. The red-billed oxpecker, African black-headed oriole, and ring-necked dove all occur throughout Eastern and Southern Africa [65, 66]. Ring-necked doves inhabit all woodlands and open tree savannas across East and Southern Africa [65]; red-billed oxpeckers occur throughout the eastern savannas of sub-Saharan Africa [66]; and African black-headed orioles are found in broad-leaved woodlands and riverine forests across Eastern and Southern Africa [66]. Field observations confirmed the presence of all three bird species across the research areas, with red-billed oxpeckers regularly observed on giraffes in each site. This supports the appropriateness of the playback stimuli and ensures consistent ecological exposure across giraffe populations (Fig. 2).

### Collecting of playback stimuli

Human observers approaching oxpeckers on foot reliably trigger alarm call responses [68, 69], allowing for experimental standardization and ensuring minimal disturbance. Following this method, we elicited oxpecker ‘ksss’ alarm calls [25] by gradually approaching giraffes hosting oxpeckers under natural conditions. The objective was to elicit these harsh sounding calls naturally by creating a subtle but increasing disturbance without immediately triggering a flight response in the giraffe. Free-ranging giraffes were followed on foot during their browsing and feeding activities, allowing them to habituate to human presence. The animals became accustomed to the observer (Anton Baotic, AB) after approximately three to four weeks. Once perched oxpeckers were detected, a gradual approach was continued while closely monitoring both the giraffes and the birds. Individuals hosting oxpeckers were approached slowly to prevent premature disturbance. As the distance to the giraffes decreased, oxpeckers typically began emitting alarm calls. Only recordings made at close range (10–15 m) were used for playback, ensuring high signal clarity and naturalistic acoustic properties. Audio recordings were stopped once the oxpecker(s) took flight. Audio recordings were conducted at ‘Adventures with elephants’ (research area A) between July and August 2023. Although primary field recordings of oxpecker alarm calls were collected in research area A, red-billed oxpecker alarm calls are acoustically stereotyped responses to immediate disturbance and are not known to vary regionally or with predator presence. Thus, the combined use of local and archived calls (see section ‘Audio recordings and editing of playback stimuli’) ensures that the stimuli represent generalizable alarm cues.

To ensure that observed behavioral responses of tested giraffes were specific to naturalistic oxpecker ‘ksss’ calls and not general responses to any auditory stimulus, we included non-alarm control stimuli from two other bird species: ‘coo’ calls from the ring-necked dove [70], and ‘plew’ whistle calls from the African black-headed oriole [71]. The call types of both bird species serve as conspecific contact calls and have not been described in the literature as eliciting vigilance responses in other species. These recordings were conducted opportunistically whenever individuals of these species were heard vocalizing nearby. Upon detecting a dove or an oriole, the bird was followed to obtain acoustic recordings from 10–20 m distance, ensuring minimal background noise and optimal signal clarity.

### Audio recordings and editing of playback stimuli

All bird calls were recorded with Sound Devices MixPre-3 II (frequency response: 10 Hz to 80 kHz) and a omnidirectional Neumann KM 183 MT microphone (modified for recording frequencies down to 10 Hz, frequency response: 10 Hz to 20 kHz), saved as .wav files (44.1 kHz, 16-bit). The microphone was fitted with a Rycote Cyclone windscreen to minimize wind noise and was hand-held or mounted on a tripod, directed towards the calling birds‘ beak. All audio files used in this study were visually inspected for clarity (minimal ambient noise) and audio artifacts (no friction sounds and overlap with calls from conspecifics or heterospecifics) by using spectrograms in Audacity version 3.3.

Playback stimuli were denoised, filtered, and normalized using Audacity and Praat (version 6.4.27) to ensure clarity and consistency. A total of 120 oxpecker calls (mean duration = 6.6 ± 0.8 s), 120 oriole calls (9.8 ± 0.1 s), and 108 dove calls (7.5 ± 0.3 s) were used, supplemented with recordings from the Xeno-Canto animal sound archive (see Additional file 2: Sound editing protocol [72–75]) and Blue Pigeons Productions UK. These single calls served as templates to create 24 playback packages, each containing three sequencese—one for each species. Each sequence contained several individual calls from a single species. Oxpecker sequences contained five ‘ksss’ calls (Additional file 3), oriole sequences contained five ‘plew’ calls (Additional file 4), and dove sequences contained six ‘coo’ calls (Additional file 5). These call numbers were chosen to approximate natural calling patterns based on field recordings, while also standardizing the total duration of playback stimuli across species. Further, the calls per sequence, as well as the order of sequences within each package, were randomly assigned to control for order effects. To ensure the acoustic structure of the calls remained intact, denoising and filtering procedures were validated against peer-reviewed protocols from previous research [76, 77] and carefully applied to minimize background noise without altering the spectral or temporal properties of the signals (see Additional file 2: Sound editing protocol [76, 77]). To confirm that acoustic structure was preserved under natural field conditions, we conducted propagation trials by playing and re-recording oxpecker, dove, and oriole sequences at 25, 50, 75, and 100 m. These distances were selected to reflect the actual playback distances recorded during trials (see ‘Playback design and procedure’), particularly given safety constraints in predator-inhabited areas where the observer remained inside a vehicle. Distances were measured with a Nikon Prostaff 1000 laser rangefinder, and microphone input was set at 70 dB. To ensure consistent stimuli conditions, playback amplitude was standardized to 85 dB SPL (Z-weighted) at 1 m using an NTI AL1 SPL meter with an NTI MiniSPL microphone. All stimuli were broadcast using a JBL-EON Compact loudspeaker (37.5 Hz-20 kHz frequency response) connected via Bluetooth to an Apple iPhone 14 Pro. Although published SPL data for red-billed oxpecker alarm calls are lacking, this level reflects the natural vocalization range of similarly sized passerines. For example, red-winged blackbirds (*Agelaius phoeniceus*) vocalize at 85–95 dB SPL [78], Grey go-away bird (*Crinifer concolor*) alarm calls were broadcast at 93–95 dB SPL [40], Orchard oriole (*Icterus spurius*) calls at 85 dB SPL [79], and doves of the genus Streptopelia at 75–80 dB SPL [80]. This ensured that playback stimuli were ecologically realistic. Re-recordings confirmed that acoustic signals remained structurally intact across all distances, validating consistent stimulus perception by giraffes during trials (spectrograms are provided in Additional file 2: Fig. S1).

### Playback design and procedure

**Fig. 3:**
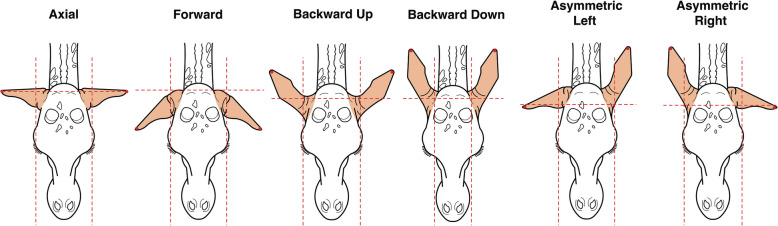
Giraffe ear postures. *Axial:* Ears extend horizontally outward, forming a straight line approximately at a 90-degree angle to the head-to-tail axis. *Forward:* Ears directed forward at an angle greater than 30 degrees from the horizontal, directed toward the front of the giraffe. *Backward Up:* Ears tilt backward at an angle greater than 30 degrees from the horizontal, positioned above the line of the neck. *Backward Down:* Ears angle backward beyond 30 degrees from the horizontal, positioned at or below the level of the neck. *Asymmetric Left/Right:* One ear (left or right) angles backward while the other ear (right or left) is positioned either forward or in the axial (outward) posture

Vigilance behavior was recorded as the primary response variable, given its established role in predator detection and risk assessment in African ungulates [32]. Vigilance responses were quantified through a set of frame-coded behavioral parameters, including latency to not feeding, orientation toward the speaker, duration of turning head and neck toward the loudspeaker, ear position, and active scanning (see below for the coding protocol; Table 5 for the full ethogram and Fig. 3 for ear positions).

**Table 5:** Descriptions of the behaviors used to analyze giraffes’ response to playback stimuli

Behavior	Description
*Feeding*	The individual engages in continuous consumption of leaves or branches with observable mouth-to-vegetation contact
*Not feeding*	The individual halts feeding behavior entirely, showing no further interaction with foliage
*Turn to*	The individual stops feeding and directs its head and neck toward the speaker or sound source. Only the first instance of head/neck orientation toward the speaker was scored per trial to quantify initial vigilance onset
*Ear posture*	The five ear positions ('Axial,''Forward,''Backward up,''Backward down,''Asymmetric left/right') are used to assess attentional states
*Scanning*	Following the ‘Turn to’ response, the individual actively moves its head to observe the surroundings. Revisiting the speaker's direction is included in this behavioral category
*Approach*	The individual walks at least three steps toward the loudspeaker’s direction. If the giraffe stops, looks attentively at the loudspeaker, ‘Scanning’ is coded
*Displacement*	The individual walks continuously for at least 10 s in the opposite direction of the loudspeaker

To isolate responses to heterospecific alarm cues and avoid potential social influence of conspecifics on vigilance behavior, only one giraffe at a time was tested, with no other giraffes present within 25 m [33]. This ensured that vigilance was attributable solely to oxpecker alarm calls, controlling for collective vigilance effects.

Giraffes were approached either on foot or by vehicle depending on field conditions and safety considerations. In all cases, trials were initiated only when the focal individual was stationary at a bush or tree, calmly feeding with its mouth in contact with foliage, and showing no signs of vigilance or distress. In the predator-inhabited reserve, where giraffes are habituated to vehicles due to regular game drives and park management activities, playback began only after the vehicle was stationary and the giraffe had resumed foraging. Individuals were selected only if they were free of perched oxpeckers and no audible vocalizations from oxpeckers, orioles, or doves were present before or during the trial. To eliminate potential confounds, no engine noise or other vehicles were present, and the speaker was hidden behind vegetation, directed toward the giraffe. GPS coordinates of the speaker and giraffe were recorded post-trial to calculate linear playback distance (range: 15.3–93.6 m). The observer (AB) remained stationary and filmed the focal individual using a Sony FDR-AX53 video camera (Fig. 1B, see also video in Additional file 1). Giraffes were identified by their unique coat patterns [81, 82] and assigned unique ID codes with photographic documentation (ID-kit) of the head, and left and right side of the body. Age classes followed established categories [83]: adults (> 4 years), subadults (< 4 years) and calves/juveniles (< 1 year).

Each playback package consisted of three sequences. The playback of each sequence consisted of three phases. After at least 30 s of continuous feeding, the (1) baseline phase recorded natural and calm feeding behavior and served as a reference. The (2) response phase followed, with a playback of the first sequence of the playback package, and concurrent behavioral observations. The (3) post-phase began once the giraffe resumed feeding or continued feeding for 10 s post-playback. This process was repeated until all sequences of the playback package were presented.

In some cases, giraffes moved into dense vegetation before all playback sequences could be completed, occasionally resulting in only one control sequence being presented. A trial was considered valid if at least one control (dove or oriole) and the oxpecker alarm call sequence were successfully presented, regardless of order. If a giraffe left before a control session could be conducted, the trial was incomplete. Re-testing occurred only if the same individual was re-identified (via ID-kit) on a different day, using a different playback package. A minimum 24-h interval separated all re-tests. If the giraffe relocated to feed elsewhere after one successful sequence, AB followed the individual until it resumed calm feeding, at which point the next sequence was presented. In total, 53 individual giraffes were tested across the three research areas. Each individual received at one oxpecker alarm call stimulus, and depending on trial conditions, one or both control stimuli (dove and oriole).

### Behavioral coding of giraffes’ responses to playbacks

Videos were coded frame-by-frame (frame = 20 ms) by AB using Solomon Coder version 17.03 for Windows. The following behavioral categories were measured: ‘Feeding’, ‘Not Feeding’, ‘Turn To’, ‘Scanning’, ‘Approach’, ‘Displacement’ (for definition see Table 5), and ‘Ear Posture’ (see illustration in Fig. 3). We used continuous sampling by measuring the duration (in seconds) of each behavior. For a table of the descriptive measures of each behavioral response see Additional file 2: Table S3. Response latency was defined as the time (in seconds) from playback onset to the first observable vigilance behavior, typically head or neck orientation toward the loudspeaker. This behavioral shift was typically accompanied by a transition from ‘Feeding’ to ‘Not Feeding,’ as coded in the ethogram Table 5). If the focal giraffe showed displacement behavior by walking continuously in the opposite direction of the loudspeaker for at least 10 s, the playback trial was considered completed. Only the first head or neck orientation toward the loudspeaker was used to define vigilance onset, from which latency was calculated. If no relevant behavior occurred within 20 s of stimulus onset, a maximum latency of 20 s was assigned by the software. These censored values indicated no detectable response and were excluded from mean latency calculations (Additional file 2: Table S4).

A subset of the videos of 18 tested individuals (32%) was blind-coded by Georgine Szipl (GS), and the behavioral measures of ‘Feeding’ during baseline, and ‘Not feeding’, ‘Ears forward’, ‘Turn to’, and ‘Scanning’ during the response phases were used to calculate intraclass corelation coefficients (ICC) in order to determine the consistency and absolute agreement between raters. ICC were calculated for each playback sequence (i.e., stimulus type) separately. The two-way random-effects model was chosen, and reliability was estimated for a single rater. Inter-rater consistency and absolute agreement showed excellent results (kappa > 0.9) for all behavioral measures compared. ICC kappa values and 95% confidence intervals are presented in Additional file 2: Table S5.

### Acoustic analysis of oxpecker ‘ksss’ calls: duration and mean HNR

Playback stimuli from the created playback packages were analyzed to obtain (a) the duration of each single call (in seconds), (b) the total duration of stimuli sequences (including inter-call intervals) per bird species per playback package, and (c) the harmonic-to-noise ratio (HNR) of single calls. HNR represents the ratio of energy in harmonic components to energy in noisy components, with lower HNR values indicating a lower proportion of harmonic energy [84]. Importantly, acoustic analyses were conducted after the playback experiments had been completed and were not used to guide stimulus selection. The HNR analysis was performed post hoc to explore whether acoustic structure may have contributed to variation in vigilance responses. Acoustic analysis was conducted using Praat. Durations, either of single calls or of the total playback package, were obtained using the ‘Get total duration’ command, while HNR was measured using the ‘To Harmonicity (cc)’ command with a time step of 0.001, a silence threshold of 0.1, and one period per window. Minimum fundamental frequency (pitch floor) was set to 450 Hz for dove and oriole calls and 720 Hz for oxpecker calls. Mean HNR was calculated by measuring the HNR of each individual call within a given sequence (excluding silent intervals between calls) and averaging these values across all calls in that sequence.

### Acoustic comparison between oxpecker ‘ksss’ calls and giraffe hisses: fpeak and duration

To address the possibility that giraffes respond to oxpecker alarm calls because they acoustically resemble giraffe hisses, which are also broadband and noisy in structure [5], we compared key acoustic parameters between the two call types. We extracted the ‘frequency at maximum amplitude’ (fpeak) and call duration from 22 randomly selected red-billed oxpecker ‘ksss’ calls recorded for this study, and compared these values with published acoustic measurements of 22 giraffe hisses reported in the supplementary materials of [5]. To ensure compatibility in analysis methods, we used the two giraffe hiss audio samples provided by Volodina et al. (2018) [5] to confirm that our approach reproduced their published values. Spectrogram settings were: view range 0–15000 Hz, window length = 0.01 s, dynamic range = 50 dB. We used default advanced spectrogram settings, except that we selected a ‘Hamming’ window shape. Fpeak was determined using the ‘view spectral slice’ function in Praat.

### Statistical analysis

We conducted a principal component analysis (PCA) to condense correlated vigilance behaviors into a smaller number of components that captured broader patterns of response. This reduced redundancy across variables and allowed us to examine how giraffes adjusted their behavior as cohesive patterns, providing a clearer and ecologically meaningful interpretation of how giraffe vigilance responses were shaped by both stimulus type and predator exposure. Five variables loaded strongly and positively onto a single principal component (hereafter PC) with an eigenvalue greater than 1.0, explaining 83.15% of the total variance: the duration of ‘Not feeding’, ‘Ears forward’, ‘Turn to’, ‘Scanning’, and ‘Total response time’. This component captured both initial reactions to stimuli (not feeding and turning toward the sound source, ears forward) and sustained alert behaviors (scanning, total response duration). Therefore, we termed this component the ‘vigilance behavior’ component. Behavioral categories with eigenvalues below 1.0 that loaded onto separate PCs were discarded. The factor loadings are provided in Table 6. The overall Kaiser–Meyer–Olkin (KMO) measure of sampling adequacy was 0.8, with estimates ranging from 0.71 to 0.92, confirming the data was suitable for PCA. We extracted regression scores of the PC for further analyses. All behavioral categories had positive loadings on the PC (cp. Table 6). Thus, higher positive regression scores translate to higher values of the behavioral categories summarized in this PC, and can roughly be interpreted as longer durations of vigilance behaviors. To confirm that playback distance did not influence giraffe vigilance behavior in response to oxpecker alarm calls, we conducted a Spearman rank correlation between speaker-giraffe distance and vigilance behavior component scores (PC1) for oxpecker trials only (ρ = −0.05, *N* = 49, *P* = 0.72), indicating that response strength was not affected by the proximity of the playback stimulus.

**Table 6:** Principal component and durations of behavioral categories. PC extracted from PCA showing standardized loadings (PC), communalities (h^2^), and uniqueness (u^2^) for behavioral response durations (in seconds) measured during playback experiments. The Kaiser–Meyer–Olkin (KMO) measure of sampling adequacy was 0.8

**Behavioral categories**	**Behavioral response duration (s)**			**PCA**		
	**Minimum**	**Maximum**	**Mean ± Standard deviation**	**PC**	**h**^**2**^	**u**^**2**^
Total response time	5	96.9	14.1 ± 12.8	0.98	0.95	0.047
Not feeding	1.8	68.5	18.5 ± 16.7	0.95	0.9	0.096
Ears forward	0.6	82.3	16.6 ± 17.9	0.91	0.83	0.171
Turn to	2	50.05	13.8 ± 10.5	0.86	0.75	0.253
Scanning	2.7	44.9	12.7 ± 9.9	0.85	0.72	0.275

We applied a Linear Mixed Model (LMM) to analyze the ‘vigilance behavior’ component, using a Gaussian distribution with an identity link function, implemented in the lme4 package [85]. To account for repeated measures of the same individuals (i.e., call sequences of the different bird species), we included giraffe identity as a random effect. Fixed effects included stimulus type (dove, oriole, oxpecker), mean HNR of the playback package, research area (A, B, C), the sex of the focal giraffe (male or female), age class (calf, subadult, adult), and presentation order of the stimuli (1st, 2nd, or 3rd). Additionally, we included an interaction term between stimulus type and research area to explore potential local environmental influences on response variation.

Multicollinearity among fixed effects was assessed prior to modelling [86]. Significant collinearity was detected between stimulus type and mean HNR of the stimuli. To address this, we run separate models: one incorporating stimulus type as a fixed effect and another incorporating mean HNR as a fixed effect. We then used Likelihood Ratio Tests (LRT) to compare the null model with each full model (either containing stimulus type or mean HNR), and the two full models with each other. LRT revealed that the full models explained variation in the data significantly better than the null model, which included only the random factor and the intercept (LRT: stimulus type: χ^2^ (13) = 90.553, *p* < 0.0001); mean HNR: χ^2^ (10) = 86.374, *p* < 0.0001). No difference was found between the full model containing the fixed effect stimulus type and the full model fitted using mean HNR (χ^2^ (3) = 4.179, *p* = 0.243), indicating that both models explained variation in the responses equally well.

Differences in response latencies were analysed using non-parametric tests. We conducted Kruskal–Wallis tests followed by Dunn’s post-hoc tests with Bonferroni adjustment, which preserve rank structure and adjust for multiple comparisons and unequal sample sizes. This choice was made to address slight differences in sample size across stimuli: oxpecker alarm calls were tested on 26 giraffes, oriole calls on 25, and dove calls on 22 individuals. Due to logistical constraints in the field, not all individuals received both control stimuli. Wilcoxon-Mann–Whitney-U (MWU) tests were used to statistically compare the oxpecker calls with the published giraffe hiss measurements.

All statistical analyses were conducted in R Studio Version 2024.12.0 + 467 [87], using the ‘psych’ package [88] for PCA, the ‘lme4’ package [85] for fitting linear mixed models, the ‘irr’ package [89] to calculate interrater reliability via the intraclass correlation coefficient (ICC) [90], the ‘emmeans’ package to conduct pairwise comparisons for the interaction terms [91], the ‘dunn-test’ package [92] for post-hoc tests of Kruskal Wallis tests, and the MWU tests using the ‘coin’ package [93].

## Supplementary Information

Additional file 1: Video file showing behavioral responses of a giraffe to playback stimuli.

Additional file 2: Table S1 Effect of stimulus type on ‘vigilance behavior’ component. Table S2 Effect of mean harmonic-to-noise ratioon giraffe vigilance behavior. Table S3 Behavioral responses of giraffes to playback stimuli. Table S4 Latency measures in response to playback stimuli. Table S5 Inter-rater reliability of behavioral measures. Figure S1 Representative spectrograms of African black-headed oriole, ring-necked dove, and red-billed oxpecker calls re-recorded at 25 m, 50 m, 75 m, and 100 m during propagation tests. Sound editing protocol for section ‘Audio recordings and editing of playback stimuli’ - description of the audio editing process, including sound selection, filtering, and standardization used to prepare playback sequences.

Additional file 3: Red-billed oxpecker playback stimulus.

Additional file 4: African black-headed oriole playback stimulus.

Additional file 5: Ring-necked dove playback stimulus.

Additional file 6: This file contains the dataset analyzed in this study.

## Data Availability

Source data are provided in Additional file 6 within this paper.

## Abbreviations

dB: Decibel
Fpeak: Frequency at maximum amplitude
HNR: Harmonic-to-noise ratio
Hz: Hertz
LMM: Linear mixed-effect model
MWU: Wilcoxon-Mann–Whitney U test
PCA: Principal component analysis
SPL: Sound pressure level
s: Seconds(s)
