# Supplementary material for: Predator experience enhances giraffe vigilance to oxpecker alarm calls
Source: BMC Biol. 2025 Oct 10;23:304. doi: 10.1186/s12915-025-02395-5 (PMC12512274; doi:10.1186/s12915-025-02395-5)
Supplement: Supplementary file 2 — Additional file 2: Table S1 Effect of stimulus type on ‘vigilance behavior’ component. Table S2 Effect of mean harmonic-to-noise ratioon giraffe vigilance behavior. Table S3 Behavioral responses of giraffes to playback stimuli. Table S4 Latency measures in response to playback stimuli. Table S5 Inter-rater reliability of behavioral measures. Figure S1 Representative spectrograms of African black-headed oriole, ring-necked dove, and red-billed oxpecker calls re-recorded at 25 m, 50 m, 75 m, and 100 m during propagation tests. Sound editing protocol for section ‘Audio recordings and editing of playback stimuli’ - description of the audio editing process, including sound selection, filtering, and standardization used to prepare playback sequences. [file 12915_2025_2395_MOESM2_ESM.pdf]

Supplementary Information

Baotic A and Szipl G. Predator experience enhances giraffe vigilance to oxpecker alarm calls. *BMC Biology* 2025

Supplementary Tables:

Supplementary Table S1 | Effect of stimulus type on 'vigilance behavior' component. Estimated means (EM), standard errors (SE), effect sizes (t values) and lower and upper confidence intervals (CI) for all coefficients in the full models fitted with linear-mixed models. The model examines the effect of stimulus type (oxpecker, dove, or oriole calls), research area, age class, sex, and stimulus type presentation order on the regression scores of the vigilance behavior component. Strong effects (bold type; CI not overlapping zero) indicate that vigilance was higher in response to oxpecker alarm calls compared to control stimuli (dove and oriole). No strong effects were found for research area, age class, sex, or playback order.

**Coefficients**	**Estimated mean**	**SE**	**t value**	**CI lower**	**CI upper**
(Intercept)	-0.503	0.187	-2.694	-0.872	-0.133
Stimulus Type					
Dove vs. Oriole[a]	-0.001	0.220	-0.005	-0.436	0.433
Dove vs. Oxpecker[a]	1.042	0.215	**4.850**	**0.618**	**1.465**
Oriole vs. Oxpecker[b]	1.043	0.208	**5.006**	**0.632**	**1.454**
Research area					
A vs. B[c]	0.021	0.234	0.088	-0.442	0.482
A vs. C[c]	-0.040	0.350	-0.114	-0.745	0.651
B vs. C[d]	-0.061	0.348	-0.174	-0.755	0.724
Age class					
Adult vs. Calf[e]	0.173	0.195	0.886	-0.217	0.561
Adult vs. Subadult[e]	-0.187	0.219	-0.856	-0.634	0.245
Calf vs. Subadult[f]	-0.360	0.260	-1.383	-0.888	0.155
Sex					
Female vs. Male[g]	0.109	0.131	0.829	-0.154	0.372
Order					
1st vs. 2nd[h]	0.119	0.148	0.807	-0.172	0.411
1st vs. 3rd[h]	-0.040	0.154	-0.258	-0.343	0.264
2nd vs. 3rd[i]	-0.159	0.161	-0.984	-0.477	0.160
Set as reference point:<br>[a] Dove, [b] Oriole, [c] research area A, [d] research area B, [e] Adult, [f] Calf, [g] Female, [h] first, [i] second					

Supplementary Table S2 | Effect of mean harmonic-to-noise ratio (HNR) on giraffe vigilance behavior. Estimated means (EM), standard errors (SE), effect sizes (t values) and lower and upper confidence intervals (CI) for all coefficients in the full model fitted with the linear-mixed model are shown. The model examines the impact of mean HNR, research area, age class, sex, and stimulus presentation order on the regression scores of the 'vigilance behavior' component. Bold type indicates coefficients with strong effects and CI not overlapping zero.

**Coefficients**	**Estimated mean**	**SE**	**t value**	**CI lower**	**CI upper**
(Intercept)	0.578	0.190	3.049	0.204	0.952
Mean HNR	-0.055	0.010	**-5.599**	**-0.074**	**-0.036**
Reseach area					
A vs. B[a]	-0.219	0.244	-0.897	-0.700	0.262
A vs. C[a]	1.563	0.350	**4.464**	**0.872**	**2.253**
B vs C[b]	1.782	0.354	**5.034**	**1.083**	**2.480**
Age class					
Adult vs. Calf[c]	0.103	0.200	0.515	-0.296	0.503
Adult vs. Subadult[c]	-0.278	0.225	-1.237	-0.736	0.167
Calf vs. Subadult[d]	-0.381	0.268	-1.419	-0.926	0.151
Sex					
Female vs. Male[e]	0.165	0.136	1.213	-0.107	0.437
Order					
1st vs. 2nd[f]	0.120	0.147	0.814	-0.171	0.411
1st vs. 3rd[f]	0.013	0.153	0.088	-0.290	0.316
2nd vs. 3rd[g]	-0.106	0.158	-0.673	-0.419	0.206

Set as reference point:
[a] research area A, [b] research area B, [c] Adult, [d] Calf, [e] Female, [f] first, [g] second

Supplementary Table S3 | Behavioral responses of giraffes to playback stimuli. Mean ± SE, minimum (Min.), and maximum (Max.) values for behaviors grouped by category (e.g., ear position, vigilance, movement), in response to dove, oriole, and oxpecker calls. Notable increases in response intensity for oxpecker stimuli are bolded.

**Behavioral Response**	**Stimulus Type**	**Mean ± SE**	**Min**	**Max**
Ears asymmetric	Dove	1.72 ± 2.55	0	7.8
	Oriole	1.86 ± 3.47	0	12.25
	**Oxpecker**	**3.65 ± 4.10**	0	15.05
Ears backward down	Dove	3.01 ± 3.52	0	10.1
	Oriole	3.97 ± 4.46	0	10.8
	Oxpecker	2.70 ± 4.21	0	19
Ears backward up	Dove	1.15 ± 2.51	0	7.45
	Oriole	1.92 ± 3.05	0	9.9
	Oxpecker	1.12 ± 2.20	0	9.65
Not feeding	Dove	0.00 ± 0.00	0	0
	Oriole	0.00 ± 0.00	0	0
	**Oxpecker**	**12.60 ± 16.24**	0	68.5
Ears forward	Dove	0.34 ± 1.42	0	7.9
	Oriole	0.18 ± 1.08	0	7.45
	**Oxpecker**	**10.45 ± 16.74**	0	82.25
Ears axial	Dove	1.18 ± 2.19	0	7.25
	Oriole	1.15 ± 2.38	0	8.8
	Oxpecker	4.84 ± 8.40	0	38.6
Scanning	Dove	0.08 ± 0.58	0	4.05
	Oriole	0.00 ± 0.00	0	0
	**Oxpecker**	**6.62 ± 9.60**	0	44.9
Turn to	Dove	0.00 ± 0.00	0	0
	Oriole	0.11 ± 0.80	0	5.6
	**Oxpecker**	**10.46 ± 10.88**	0	50.05
Total response time	Dove	8.05 ± 2.86	6.8	23.25
	Oriole	9.95 ± 0.67	6.75	12.85
	**Oxpecker**	**23.11 ± 17.46**	5	96.85
Approach	Dove	0.00 ± 0.00	0	0
	Oriole	0.00 ± 0.00	0	0
	Oxpecker	0.93 ± 2.83	0	12.8
Displacement	Dove	0.21 ± 1.44	0	10
	Oriole	0.00 ± 0.00	0	0
	Oxpecker	0.76 ± 2.47	0	9.2
Bolded Oxpecker values indicate marked behavioral changes compared to Dove and Oriole stimuli (especially in Not feeding, Ears forward, Scanning, Turn to, Total response time).				

Supplementary Table S4 | Latency measures in response to playback stimuli. Mean ± SE, Min., and Max. values (in seconds) for latency to not feeding and orient toward the speaker in response to dove, oriole, and oxpecker calls.

Stimulus type	Latency to not feeding (s)				Latency to turn to (s)			
	Mean	SE	Min.	Max.	Mean	SE	Min.	Max.
Dove	20	0	20	20	20	0	20	20
Oriole	20	0	20	20	19.62	2.69	1.15	20
Oxpecker	10.1	8.17	0.25	20	7.11	7.7	0.2	20

Supplementary Table S5 | Inter-rater reliability of behavioral measures. Intra-class correlation coefficients (for absolute agreement and consistency) with 95% confidence intervals (CI) between two raters for measured behaviors of each playback session (1st-3rd) that were summarized in the principal component. N denotes the number of individuals analyzed.

Behavioural variable	Absolute agreement	95% CI		Consistency	95% CI		N
		Lower	Upper		Lower	Upper	
Feeding Baseline (1st)	1	1	1	1	1	1	18
Feeding Baseline (2nd)	1	1	1	1	1	1	19
Feeding Baseline (3rd)	1	1	1	1	1	1	14
Ears forward (1st)	0.923	0.814	0.969	0.923	0.812	0.97	19
Ears forward (2nd)	0.992	0.979	0.997	0.992	0.979	0.997	17
Ears forward (3rd)	0.992	0.979	0.997	0.992	0.979	0.997	17
Turn to (1st)	0.999	0.997	1	0.999	0.997	1	19
Turn to (2nd)	1	1	1	1	1	1	17
Turn to (3rd)	1	1	1	1	1	1	14
Not feeding (1st)	0.959	0.899	0.984	0.959	0.898	0.984	19
Not feeding (2nd)	0.996	0.99	0.999	0.996	0.989	0.999	17
Not feeding (3rd)	0.993	0.98	0.998	0.993	0.98	0.998	14
Scanning (1st)	0.988	0.971	0.996	0.989	0.972	0.996	19
Scanning (2nd)	0.996	0.988	0.998	0.996	0.988	0.998	17
Scanning (3rd)	0.998	0.995	0.999	0.998	0.995	0.999	14

Supplementary Figure:

Supplemental Figure S1 | Spectrograms of playback stimuli types. Representative spectrograms of African black-headed oriole, ring-necked dove, and red-billed oxpecker calls, re-recorded at 25 m, 50 m, 75 m, and 100 m during propagation tests (FFT window = 0.02 s, dynamic range = 70 dB).

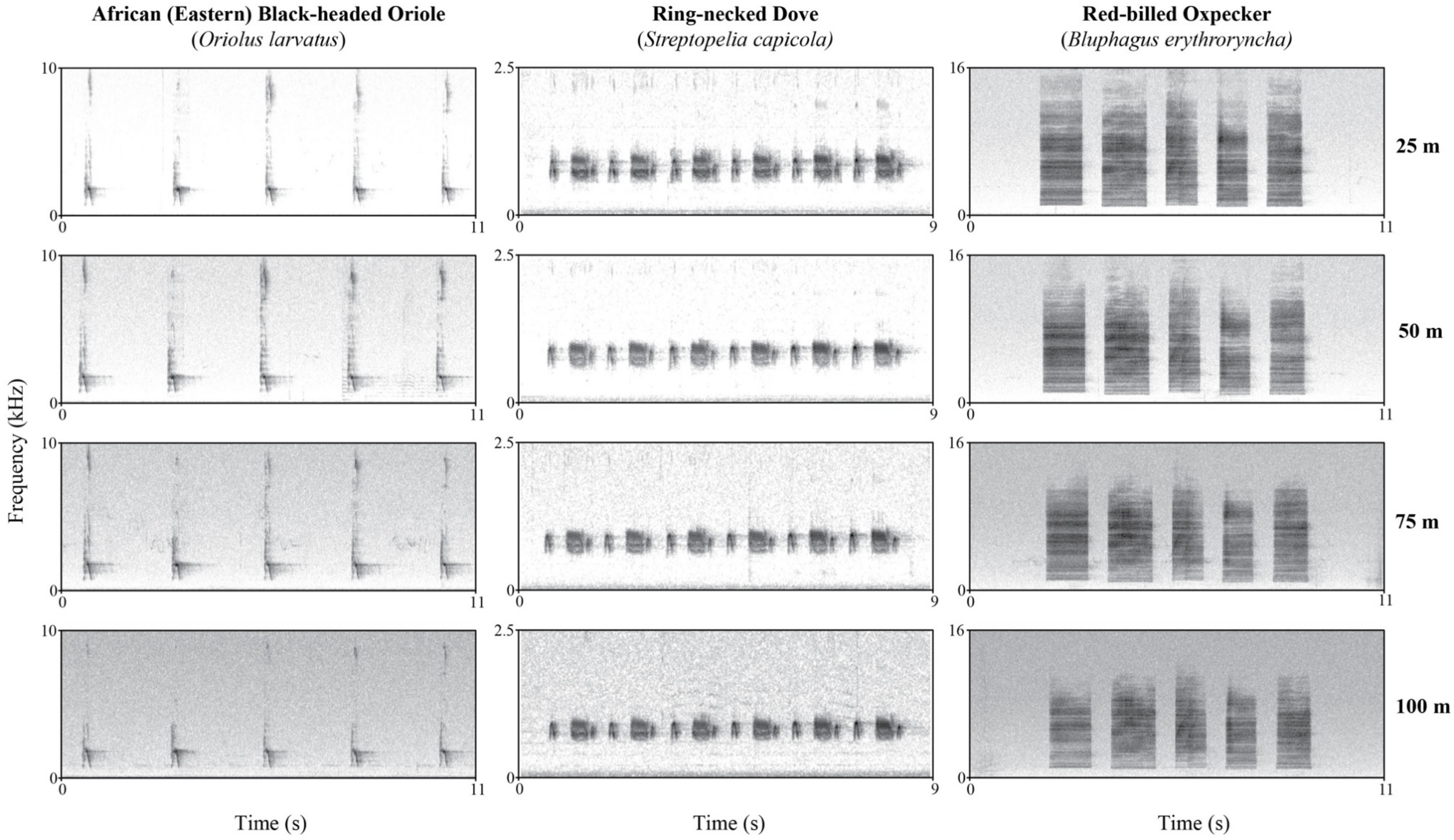

Sound editing protocol:

Section “Audio recordings and editing of playback stimuli” - description of the audio editing process, including sound selection, filtering, and standardization used to prepare playback sequences:

We retained field recordings of 90 single oxpecker ‘ksss’ calls, 108 ‘coo’ calls from ring-necked doves, and 105 ‘plew’ calls from African black-headed orioles. To ensure a sufficient and well-balanced sample size, additional calls were incorporated from external sources while maintaining consistency in acoustic characteristics between recorded and sourced calls. Oxpecker calls were supplemented with 10 calls from recording XC590464 [72], 12 calls from XC718396 [73], and 8 calls from XC718397 [74] from the Xeno-Canto sound archive. Five oriole calls from XC590463 [75] were included, along with 10 additional oriole calls provided by Blue Pigeons Productions UK. The same processing procedure was applied to these external calls, resulting in a final dataset of 120 oxpecker, 120 oriole, and 108 dove single calls. Since the number of dove calls was lower than the other species, some dove tracks were used more than once to balance the dataset.

To minimize ambient noise in the playback signals, we applied the ‘noise reduction’ effect in Audacity. We selected this method because noise profile filtering has been shown to effectively reduce background noise while preserving the acoustic properties of vocalizations [76, 77]. A one-second noise profile was created before the onset of a call, followed by a 4-10 dB noise reduction (depending on the recording’s acoustic characteristics), using a sensitivity setting of 6.0 and three frequency smoothing bands. To ensure consistent peak amplitude across all sequences, while avoiding distortion due to excessive loudness (clipping effect), we normalized the audio files to -1.0 dB peak amplitude using Audacity.

To further enhance sound clarity, additional spectral content filtering was performed in Praat version 6.3.10. A high-pass filter was applied to remove excess low-frequency background noise below 450 Hz for dove and oriole calls, and below 720 Hz for oxpecker calls. A low-pass filter was used to remove upper ambient frequencies, cutting off above 1.3 kHz in dove calls and above 20 kHz in oriole calls. For oxpecker calls, the low-pass cutoff varied, with frequencies above either 16 kHz or 20 kHz being removed depending on the recording. A final smoothing function at 100 Hz was applied to ensure clear, artifact-free playback stimuli.

Supplementary References (also included in the main publication reference list):

72. Elliott S. XC590464. Accessible at www.xeno-canto.org/590464. 1995.
73. Rudman L. XC718396. Accessible at www.xeno-canto.org/718396. 2022.
74. Rudman L. XC718397. Accessible at www.xeno-canto.org/718397. 2022.
75. Elliott S. XC590463. Accessible at www.xeno-canto.org/590463. 1995.
76. Baker MC, Logue DM. A comparison of three noise reduction procedures applied to bird vocal signals. *J Field Ornithol* 2007, 78(3):240-253.
77. Benedict L, Hardt B, Dargis L. Form and function predict acoustic transmission properties of the songs of male and female canyon wrens. *Front Ecol Evol* 2021, 9.
